# Tobacco and Nicotine Use Among Active Component U.S. Military Service Members: A Comparison of 2018 Estimates from the Health Related Behaviors Survey and the Periodic Health Assessment

**Published:** 2024-03-20

**Authors:** James D. Mancuso, Anwar E. Ahmed, Kristen R. Rossi

**Affiliations:** 1 Uniformed Services University of the Health Sciences Department of Preventive Medicine and Biostatistics; 2 Armed Forces Health Surveillance Division, Defense Health Agency

## Abstract

This study compared estimates of the prevalence of and risk factors for tobacco and nicotine use obtained from the 2018 Health Related Behaviors Survey (HRBS) and Periodic Health Assessment (PHA) survey. The HRBS and the PHA are important Department of Defense sources of data on health behavior collected from U.S. military service members. While their collection methods differ, some survey questions are similar, which provides an opportunity to compare survey estimates. Active duty service members consistently reported a much lower prevalence of all types of tobacco and nicotine use on the PHA compared to the HRBS: cigarettes (11.1% vs. 18.4%), e-cigarettes (7.3% vs. 16.2%), chewing tobacco (9.7% vs. 13.4%), any tobacco or nicotine use (25.3% vs. 37.8%), and use of 2 or more tobacco or nicotine products (5.8% vs. 17.4%). Associations between tobacco and nicotine use as well as demographic and other behavioral variables were fairly similar, including age, sex, education, race and ethnicity, rank, and alcohol use. The associations with service branch, body mass index, and sleep were inconsistent. This results of this study suggest that the PHA can provide timely information on trends in military tobacco and nicotine use over time, but much higher estimates from the confidential, voluntary HRBS reported in this study suggest that the command-directed PHA may substantially underestimate the prevalence of all types of tobacco and nicotine use.

**What are the new findings?:**

U.S. service members consistently reported much lower prevalence of use of tobacco and nicotine on the PHA compared to the HRBS. The command-directed PHA may provide timely information on trends and risk factors for tobacco and nicotine use but substantially underestimates their use.

**What is the impact on readiness and force health protection?:**

The use of tobacco and nicotine remains an important threat to the health and readiness of U.S. military service members. Both the HRBS and the PHA provide unique and valuable information for military policy guidance on force health protection and readiness, and they should be used in tandem.

## 1 BACKGROUND

The HRBS is a confidential, cross-sectional survey sponsored by the Department of Defense (DOD) for better understanding of the health behaviors of all military branches. The HRBS was most recently conducted in 2018 by the RAND Corporation (Santa Monica, CA). Despite its utility in monitoring health-related behaviors in the U.S. military over time, the HRBS is limited by a low response rate, increasing the probability of bias.^1^

The periodic health assessment (PHA) is an annual, standardized health assessment throughout the military services to “assess currency of individual medical readiness (IMR) requirements,” in accordance with DOD Instructions 6025.19 and 6200.06.^2,3^ The PHA collects some survey items similar to the HRBS, including questions on smoking and tobacco and nicotine product use.^3^ As the PHA must be reviewed by a health care provider, often in a direct, personal encounter,3 its data may not be reported accurately due to service member concerns about negative career consequences, or other social desirability misclassification biases.^4^

In 2015 the prevalence of cigarette use reported in the HRBS within the active duty population (13.8%) declined for the first time to a point lower than the general U.S. population (15.1%).^5,6,7,8^ In contrast, the prevalence of electronic cigarette (e-cigarette) use—also known as vaping—was higher in the U.S. military (12.4%) than in the general U.S. population (3.5%).^5,9^ Particularly notable was the much higher prevalence of e-cigarette use among the 17-24 year old age group in the military (22.8%) compared to the general population (5.2%). The most recently published HRBS results from 2018 indicate substantial increases in overall cigarette use (18.4%) and e-cigarette use (16.2%) compared to 2015.1

A comparison of these 2 data sources could be helpful in determining the validity and utility of both data sources both for surveillance purposes and public health guidance. Furthermore, such a comparison could obviate the need for questions in the HRBS that are already covered in the PHA, if the 2 data sources are found to provide similar information.^1^ This study compares estimates of prevalence and risk factors for tobacco and nicotine use among active duty U.S. service members that were self-reported in 2018 in the HRBS and the PHA.

## 2 METHODS

The 2018 HRBS study design included population-based stratified random sampling and non-responsive weights, which were utilized to make the analytic sample obtained from the study representative of the eligible service member population.^1^ Publicly available data from the 2018 HRBS, the most recent data released, were utilized to create a sampling frame of 1,357,219 active component service members (ACSMs), which was segmented into 50 strata, based on the interaction of service branch (5 categories), pay grade (5 categories), and sex. Of 199,996 invited eligible active duty service members, 17,166 responded to the survey request, with an overall weighted response rate of 9.6%.^1^ Sampling weights by post-stratification were used to represent the population. The low response rate of HRBS increases risk of selection bias and bias due to unobserved data. To increase representativeness, HRBS used SAS 9.4 to produce summary statistics, with confidence interval (CI) estimates computed using the Wald method.^1^ Missing data among respondents was addressed via imputation methods such as predictive mean matching to impute binary, ordinal, and continuous variables, and polytomous regression to impute categorical data.^1^

All PHAs completed (n=854,579) during calendar year 2018 by ACSMs in the Army, Navy, Air Force, Marine Corps, and Coast Guard were queried from the Defense Medical Surveillance System (DMSS).

HRBS and PHA questions assessing tobacco and nicotine use, demographic characteristics, and other exposures were reviewed. The questions that assessed tobacco and nicotine use were very similar, as shown in **Table 1**. Tobacco and nicotine use outcomes were assessed for 5 types of single product use within the past 30 days: cigarettes, chewing tobacco or snuff, cigars (cigars, cigarillos, or little cigars), tobacco use in a pipe or hookah, and electronic cigarettes (‘e-cigarettes’ or ‘vaping’). The use of any tobacco or nicotine product use and 2 or more forms of any single tobacco or nicotine product use were also assessed.

In both surveys, demographic information (age, sex, race and ethnicity, branch of service, rank, education); body mass index (BMI); and health-related behaviors such as sleep (average hours of sleep in a 24-hour period over the last 30 days) and alcohol use (usual number of drinks containing alcohol on day[s] the service member drank in the last 30 days) were collected similarly. The assessment of sexually-transmitted infection (STI) risk defined by the HRBS included self-reporting of an STI (such as gonorrhea, syphilis, chlamydia, HPV, or genital herpes) within the past year. In contrast, the PHA defined STI risk by 1 or more of the following: a new sex partner in the past 3 months; more than 1 sex partner in the last 12 months; sexually active women less than 25 years of age; inconsistent use of latex condoms; men who have sex with men; sexual contact with person(s) with known STIs or risk of STI; exchanged money or drugs for sex; or injected drug use.

HRBS estimates were generated using sample weights to generate representative active component population estimates, as previously reported,^8^ while PHA data were reported using unweighted estimates. Relative frequency analyses were performed to describe the distribution of respondents’ demographics and health behaviors in both surveys. The prevalence estimates of tobacco or nicotine use classification groups were reported. The standard errors and CIs for weighted HRBS estimates were computed using the Taylor series method. Adjusted odds ratios (aORs) of tobacco or nicotine product use and 95% CIs were calculated using logistic regression, reported separately for the HRBS and PHA; sample weights were again used for HRBS (i.e., weighted logistic regression) but not PHA analysis. Statistical significance was set at 0.05 for all statistical tests. SAS statistical software 9.4 (SAS Institute, Cary, NC) was used in all analyses.

## 3 RESULTS

A total of 17,166 (9.6% weighted response among those invited) and 854,579 (64% of the active component population) respondents from the HRBS and the PHA, respectively, were included in the analysis (**Table 2**). PHA response distributions compared to the HRBS were lower for respondents who were: under age 25; of junior or enlisted ranks; in the Navy or Marine Corps; overweight; and with education levels of high school or less. Notably, compared with PHA respondents, HRBS respondents reported shorter sleep duration (5 hours or less per night: 30.3% vs. 11.3%) and more frequent alcohol use (3 or more times per week: 30.2% vs. 15.4%).

Service members consistently reported a much lower prevalence of all types of tobacco and nicotine use in the PHA than in the HRBS (**Table 3a, 3b, 3c**), for: cigarettes (11.1% vs. 18.4%), e-cigarettes (7.3% vs. 16.2%), cigars (2.8% vs. 10.0%), pipes or hookahs (1.5% vs. 5.2%), and chewing tobacco (9.7% vs. 13.4%). PHA estimates were also lower for any tobacco or nicotine use (25.3% vs. 37.8%) and use of 2 or more tobacco or nicotine products (5.8% vs. 17.4%).

Tobacco and nicotine use associations with demographic and behavioral variables were mostly similar between the 2 data sources. Prevalence for all types of tobacco and nicotine use was highest in youngest service members and decreased with age; of note, prevalence among 17 to 24-year-olds was much higher in the HRBS for both cigarette use (23.1% vs. 13.7%) and e-cigarette use (27.9% vs. 12.5%). Prevalence of all types of tobacco or nicotine use was higher among men than women, except for pipe and hookah use, which was higher among women. Service members who were enlisted, at increased STI risk, used more alcohol, or had lower education levels had generally a higher prevalence of all types of tobacco or nicotine use.

Non-Hispanic Black service members had the lowest prevalence of cigarette, e-cigarette, and chewing tobacco use but highest prevalence of pipe or hookah use (8.0%) and high levels of cigar use (10.2%). Hispanic service members had the highest prevalence of e-cigarette (17.3%) use and high levels of cigarette use (18.1%). These findings were generally consistent between the data sources.

Some associations between demographic and behavioral factors and tobacco or nicotine use were inconsistent between the 2 data sources. Among the services, Marines had the highest use of all types of tobacco or nicotine, but otherwise associations between services varied by type of product used and the data source. For example, prevalence of cigarette use among Navy service members (20.4%) was higher than among Army service members (18.0%) according to HRBS data, but the relationship was reversed for the PHA—estimated prevalence in the Navy (10.1%) was lower than in the Army (14.6%). Associations of hours of sleep per night were also inconsistent with some types of tobacco or nicotine use. Much of the apparent “underweight” BMI heterogeneity between the 2 data sources was likely due to the small numbers of HRBS respondents, leading to unstable estimates.

**Table 4a, 4b, 4c** shows the relationship between each demographic and behavioral factor and the different types of tobacco or nicotine use after adjusting for all other factors, which resulted in generally similar associations as seen in **Table 3a, 3b, 3c**. HRBS data demonstrated statistically significant decreases in adjusted odds of any tobacco or nicotine use among service members who were female, older, officers, with higher education levels, had more hours of sleep per night, of a race or ethnicity other than Non-Hispanic White, and with lower alcohol use levels. The findings from PHA data were similar, but with even stronger negative associations between any use and female sex, service, officer rank, and increased education level. In contrast, negative associations of slightly lower magnitude were seen in the PHA data for increasing age, while positive associations of slightly lower magnitude were seen for alcohol use.

## 4 DISCUSSION

Service members consistently reported much lower prevalence for all types, and combinations, of tobacco or nicotine use in the PHA compared to the HRBS. As reported elsewhere, HRBS data trends from 2015 to 2018 indicate increased prevalence of cigarette use (13.8% to 18.4%) and e-cigarette use (12.4% to 16.2%) in the U.S. military.^5,1^ Highest prevalence for both types of tobacco or nicotine use was among 17-24 year olds, whose cigarette use increased from 19.3% to 23.1%, and e-cigarette use rose from 22.8% to 27.9%.^5^ Factors demonstrating generally strong associations with most or all types of increased tobacco and nicotine use included younger age, male sex, enlisted rank, lower education level, greater amounts of alcohol use, increased STI risk, and Marine Corps service. While associations between tobacco or nicotine use and demographic and behavioral variables were mostly similar in the 2 surveys, there were some inconsistent associations with branch of service.

The HRBS-obtained prevalence of cigarette use in the U.S. military was lower in 2015 than the prevalence in the general U.S. population reported by the U.S. Centers for Disease Control and Prevention (CDC),^5,9^ but HRBS-obtained prevalence then increased in 2018 to exceed the U.S. population (18.4% vs. 13.7%, respectively).^10^ In contrast, the 2018 prevalence of cigarette use in the U.S. military estimated from PHA data (11.1%) was lower than U.S. population prevalence. As the methods employed in obtaining HRBS data were more similar to those used by the CDC for civilian data than in PHA data capture, HRBS data were preferentially utilized for the remainder of military and civilian comparisons. The prevalence of e-cigarette use also remained much higher in the U.S. military than in the U.S. population (16.2% compared to 3.2%),^10^ as in 2015.^5^ “Any smoking” was higher among service members than in the U.S. population (37.8% vs. 19.7%), as was use of 2 or more tobacco or nicotine products (17.4% vs. 3.7%).^9^

Among 17-24 year olds in the U.S. military there was a much higher prevalence (23.1%) of cigarette use found than in the U.S. 18-24 year old population (7.8%).^10^ The U.S. military also had a much higher prevalence of e-cigarette use in the 17-24 age group (27.9% vs. 7.6% in the U.S. population). Any smoking was higher among 17-24 year old service members than in the U.S. population (45.7% vs. 17.1%), as was use of 2 or more tobacco or nicotine products (25.7% vs. 4.1%). The prevalence of e-cigarette use among U.S. high school students (20.8%) in 2018 was closer to the prevalence among the youngest U.S. service members (27.9%), compared to the U.S. population of the same age (7.6%), but U.S. high school student cigarette use (8.1%) was much lower than cigarette use by youngest U.S. service members (18.4%).^11^

The findings in this study are generally similar to the findings of the 2015 HRBS, but the differences between the military and civilian populations are of greater magnitude.^5^ The 7.3% prevalence estimate of e-cigarette use among ACSMs from the PHA data in this study was similar to the 9% prevalence among active and reserve service members previously reported, also using 2018-2019 PHA data.^12^ Similar to this study, in 2018 the prevalence of e-cigarette use among U.S. Air Force recruits increased to 15.3%, although prevalence of cigarette and other tobacco or nicotine product use decreased in that study but increased in this study.^13^ This study also found similar factors associated with higher prevalence of tobacco or nicotine use, as reported in previous military studies, including younger age, male sex, enlisted rank, lower education levels, greater alcohol use, and Army or Marine Corps service.^5,14^ Similar associations have also been found among the general U.S. population.^10^10

The most important strengths of this study are the large sample sizes, multiple data sources to assess the burden of tobacco and nicotine use in the U.S. military, and the comparability between the 2 sources due to similar survey questions. The stratified random survey design of the HRBS is also a contributing strength, as it provides estimates representative of the entire U.S. military population.

This work also has several important limitations. Tobacco and nicotine use have been highly dynamic in both the U.S. general and military populations in recent years, and trends have likely changed since 2018; the release of the 2024 HRBS should allow further assessment of these trends. The weighted response rate for the 2018 HRBS was low (9.6%), which could introduce selection bias into these estimates if participating service members differed from those not participating^1^1; this type of volunteer bias in survey literature generally leads to healthier participants, however, so tobacco and nicotine use estimates obtained through HRBS data utilization would have been expected to underestimate prevalence, not overestimate it.^15^15 Likewise, the 64% of service members for whom PHA data were available may have differed from those for whom data were unavailable, but because how those service members differed is unknown, the impact of this difference on prevalence estimates is unknown.

A more important source of PHA underestimation is introduction of misclassification bias if participants did not provide accurate information on their tobacco and nicotine use, which could occur due to social desirability bias, perceived stigma of tobacco and nicotine use, or other perceived negative consequences of divulging tobacco and nicotine use behaviors to other military personnel during a required military exam documented on an official form.^16^ The much lower prevalence for all types of tobacco and nicotine use in PHA data compared to the HRBS suggests this misclassification may lead to substantial bias and underestimates of the burden of tobacco and nicotine use.

Some misclassification may be non-differential; for example, the smaller aORs for age and alcohol use using PHA data compared to HRBS data suggest bias towards the null and possible non-differential misclassification. The more extreme aORs in the PHA data for other variables such as Naval service, sex, rank, and education compared to HRBS data do suggest possible differential misclassification, which could occur if service members who were female, officers, higher educated, and serving in the Navy were more affected by social desirability, stigma, or perceived negative consequences due to reporting tobacco and nicotine use.^4^ The assessment of other health behaviors such as alcohol use, drug use, and sexual practices may also be biased (particularly in the PHA) due to perceived stigma and potential for negative career consequences.^17^ The magnitude of this misclassification bias may be associated with the amount of stigma and potential consequences perceived for each behavior, so further research should consider and study their possible associations.

Tobacco and nicotine use remain an important threat to the health of U.S. military service members, resulting not only in short- and long-term health consequences, but also billions of dollars in health care and lost productivity costs,^8^ decreased fitness,^18^ and higher rates of premature discharge.^19^ Military tobacco and nicotine control policies and interventions must be guided by accurate and timely surveillance to ensure interventions are effective and responsive to dynamic societal, cultural, and economic forces that affect tobacco and nicotine use.

This study suggests that the PHA can provide timely information on trends in military tobacco and nicotine use over time, but much higher estimates in this study obtained from the confidential, voluntary HRBS also suggest that the command-directed PHA, which is part of a service member’s permanent record, may substantially underestimate the prevalence of all types of tobacco and nicotine use. Additionally, the more extreme differences between some types of service members suggest that this misclassification may be differential. This issue could potentially result in biased, and thereby invalid, associations with these demographic and behavioral risk factors, such as service members who are female, officers, higher educated, or serve in the Navy.

These differences also suggest that HRBS tobacco and nicotine use questions should not be discontinued, as some have suggested,^1^ since its data may be more valid than the PHA. The HRBS, on the other hand, suffers from a lack of timeliness, as it is only performed every 3 to 6 years, with its data lagging several years. Both data sources provide uniquely valuable information to guide military force health protection and readiness policy, and they should be used in tandem. Further research is needed to assess the validity of PHA data, not only for tobacco and nicotine use but other important health behaviors and outcomes as well.

## Figures and Tables

**Table 1.** Comparison of PHA and HRBS Smoking and Tobacco Product Use Questions

**Tobacco Use Classification**	**PHA Question**	**HRBS Question**
	**In the past 30 days, which of the following products have you used on at least 1 day…**	**On how many of the past 30 days did you…**
Cigarettes	Cigarettes?	Smoke a cigarette?
Chewing tobacco	Chewing tobacco, snuff or dip?	Use chewing tobacco or snuff?
Cigars	Cigars, cigarillos or little cigars?	Smoke cigars, cigarillos, or little cigars?
Hookah/pipe	Hookahs or waterpipes?	Smoke tobacco in a pipe or hookah?
	Pipes filled with tobacco?	
E-cigarettes	Electronic cigarettes, e-cigarettes, or vape pens?	Use electronic cigarettes, e-cigarettes, or 'vaping'?
Any tobacco product^a^	Cigarettes?; chewing tobacco, snuff or dip?; cigars, cigarillos or little cigars?; hookahs or waterpipes?; pipes filled with tobacco?; electronic cigarettes, e-cigarettes, or vape pens?; bidis?; snus?; dissolvable tobacco products?	Smoke a cigarette?; use chewing tobacco or snuff?; smoke cigars, cigarillos, or little cigars?; smoke tobacco in a pipe or hookah? use electronic cigarettes, e-cigarettes, or 'vaping'?
Two or more tobacco products	*2 or more positive responses to the cigarette, chewing tobacco, cigar, hookah / pipe, or e-cigarette classifications described above	

Abbreviations: PHA, Periodic Health Assessment; HRBS, Health Risk Behavior Survey; e-cigarette, electronic cigarette.

^a^PHA tobacco use categories for bidis, snus, and disosolvable tobacco products did not have a directly comparable terminology group for HRBS; thus, these items were not assessed for individual tobacco use comparison but were included for the 'any tobacco product' PHA classification.

**Table 2.** Demographic Characteristics of PHA and HRBS, Active Duty U.S. Military Respondents, 2018

	**PHA (n=854,579)**		**HRBS (n=17,166)**			
					95% CI	
	No.	%	Unweighted No.	Weighted %	Lower	Upper
**Sex**						
Male	705,867	82.60	11,813	83.31	82.62	83.99
Female	148,712	17.40	5,353	16.69	16.01	17.38
**Age**						
17-24	302,449	35.40	3,642	37.77	36.45	39.10
25-34	353,314	41.30	6,467	39.93	38.77	41.08
35-44	164,001	19.20	5,311	18.33	17.68	18.98
45+	34,812	4.10	1,746	3.97	3.72	4.23
Unknown	3	0.00	-	0.00		
**Service**						
Army	327,120	38.30	3,646	34.50	33.17	35.78
Navy	143,302	16.80	3,675	24.40	23.27	25.44
Marine Corps	82,705	9.70	2,569	13.90	13.14	14.66
Air Force	278,448	32.60	5,579	24.10	23.30	24.88
Coast Guard	23,004	2.70	1,697	3.20	2.98	3.36
**Rank**						
E1-E4	331,398	38.80	4,444	42.40	41.10	43.70
E5-E6	265,521	31.10	4,585	29.80	28.76	30.84
E7-W5	101,576	11.90	3,125	11.30	10.79	11.85
O1-O3	94,865	11.10	2,469	10.10	9.61	10.66
O4+	61,219	7.20	2,543	6.30	6.04	6.65
**Rank group**						
Enlisted	698,495	81.70	12,154	83.50	82.89	84.15
Officer	156,084	18.30	5,012	16.50	15.85	17.11
**STI risk**						
No	797,367	93.30	15,684	90.80	90.15	91.55
Yes	43,727	5.10	521	3.40	2.93	3.85
Unknown	13,485	1.60	961	5.80	5.21	6.32
**Sleep**						
5 hours or less	96,925	11.30	4,563	30.30	29.11	31.45
5 to less than 7 hours	464,632	54.40	5,903	33.70	32.53	34.83
7-9 hours	283,229	33.10	6,342	33.60	32.45	34.70
9 hours or more	7,005	0.80	358	2.50	2.05	2.88
Unknown	2,788	0.30	-	0.00		
**Alcohol use**						
No response / none	267,419	31.30	4,380	29.00	27.79	30.15
1-2	455,415	53.30	8,416	40.80	39.68	41.99
3-4	112,974	13.20	3,124	19.20	18.31	20.18
5-6	15,274	1.80	864	7.10	6.38	7.84
7-9	2,412	0.30	186	1.70	1.36	2.13
10+	1,085	0.10	196	2.10	1.64	2.56
**BMI group**						
Under weight	5,591	0.70	109	0.70	0.41	0.94
Normal weight	263,262	30.80	5,825	34.90	33.68	36.10
Over weight	382,743	44.80	8,761	50.00	48.76	51.21
Obese	125,271	14.70	2,471	14.50	13.63	15.27
Unknown	77,712	9.10	-	0.00		
**Education**						
High school or less	504,935	59.09	7,990	64.40	63.39	65.42
Some college	126,468	14.80	2,625	12.80	12.19	13.46
Bachelors degree or more	208,288	24.37	6,301	21.60	20.87	22.35
Unknown	14,888	1.74	250	1.20	0.90	1.42
**Race and ethnicity**						
Non-Hispanic White	490,454	57.39	10,666	57.60	56.41	58.86
Non-Hispanic Black	134,946	15.79	2,226	16.20	15.21	17.14
Hispanic	132,703	15.53	2,459	16.00	15.06	16.92
Non-Hispanic Other	96,476	11.29	1,747	9.50	8.83	10.14
Unknown	0	0.00	68	0.70	0.40	1.03

Abbreviations: PHA, Periodic Health Assessment; HRBS, Health Risk Behavior Survey; CI, confidence interval; No., number; STI, sexually-transmitted infection; BMI, body mass index.

**Table 3a.** Prevalence of Tobacco Product Use,^a^ HRBS Versus PHA, by Demographic Characteristics, Active Duty U.S. Military, 2018

	**Cigarettes**						**E-Cigarettes**						**Cigars**					
	**HRBS**				**PHA**		**HRBS**				**PHA**		**HRBS**				**PHA**	
			95% CI						95% CI						95% CI			
	No.	%^b^	Lower	Upper	No.	%	No.	%^b^	Lower	Upper	No.	%	No.	%^b^	Lower	Upper	No.	%
**Total**	2,275	18.4	17.3	19.4	94,731	11.1	1,828	16.2	15.2	17.3	62,541	7.3	1,425	10.0	9.2	10.7	23,956	2.8
** Sex**																		
Male	1,694	19.5	18.3	20.7	84,078	11.9	1,316	17.1	15.8	18.3	55,869	7.9	1,196	11.0	10.1	11.9	22,532	3.2
Female	581	12.8	11.5	14.1	10,653	7.2	512	12.0	10.5	13.5	6,672	4.5	229	4.6	3.8	5.4	1,424	1.0
** Age**																		
17-24	680	23.1	20.9	25.4	41,471	13.7	907	27.9	25.5	30.2	37,812	12.5	364	11.7	10.0	13.4	10,633	3.5
25-34	869	17.4	16.0	18.8	35,791	10.1	597	11.3	10.2	12.5	18,994	5.4	522	9.3	8.3	10.3	8,147	2.3
35-44	609	13.2	12.0	14.3	15,719	9.6	287	6.0	5.1	6.8	5,360	3.3	408	8.3	7.4	9.2	4,098	2.5
45+	117	6.8	5.4	8.2	1,750	5.0	37	1.9	1.2	2.6	374	1.1	131	7.5	6.0	8.9	1,078	3.1
** Service**																		
Army	469	18.0	15.9	20.2	47,810	14.6	246	13.9	11.7	16.1	23,581	7.2	262	8.6	7.1	10.1	10,353	3.2
Navy	540	20.4	18.2	22.7	14,409	10.1	350	17.4	15.1	19.7	10,327	7.2	341	12.2	10.3	14.0	3,396	2.4
Marine Corps	484	27.7	24.9	30.4	12,501	15.1	347	22.6	19.8	25.4	7,993	9.7	320	14.1	12.1	16.1	3,004	3.6
Air Force	579	11.9	10.9	13.0	18,521	6.7	706	14.9	13.7	16.0	19,264	6.9	358	7.1	6.3	7.9	6,573	2.4
Coast Guard	203	14.0	12.0	16.1	1,490	6.5	179	14.9	12.6	17.2	1,376	6.0	144	10.8	8.9	12.6	630	2.7
** Rank**																		
Enlisted	2,058	21.0	19.7	22.2	92,254	13.2	1,722	18.9	17.7	20.2	61,367	8.8	1,015	10.1	9.2	11.0	19,438	2.8
Officer	217	5.1	4.3	6.0	2,477	1.6	106	2.5	1.9	3.1	1,174	0.8	410	9.3	8.3	10.4	4,518	2.9
** STI risk**																		
No	2,054	18.3	17.2	19.4	86,441	10.8	1,605	15.7	14.6	16.8	56,138	7.0	1,293	9.9	9.1	10.7	21,601	2.7
Yes	102	23.0	17.1	28.9	7,042	16.1	103	24.8	18.5	31.2	5,575	12.7	52	11.3	6.7	15.9	2,195	5.0
** Sleep**																		
5 hours or less	857	26.4	24.1	28.6	17,556	18.1	605	21.8	19.5	24.1	9,745	10.1	439	12.6	10.9	14.4	3,943	4.1
5 to less than 7 hours	750	16.3	14.7	17.9	53,793	11.6	622	14.9	13.3	16.6	35,675	7.7	513	9.6	8.4	10.8	13,796	3.0
7-9 hours	614	13.3	11.7	14.9	22,691	8.0	549	12.4	10.9	13.9	16,643	5.9	450	8.1	7.0	9.2	6,054	2.1
9 hours or more	54	16.8	10.6	23.0	685	9.8	52	18.1	11.3	25.0	477	6.8	23	7.3	2.7	11.9	161	2.3
** Alcohol use^c^**																		
No response / none	389	12.3	10.4	14.1	22,580	8.4	376	13.0	11.0	15.0	19,850	7.4	158	5.0	3.7	6.3	5,069	1.9
1-2	830	13.6	12.4	14.9	44,205	9.7	629	11.2	9.9	12.5	26,784	5.9	673	9.4	8.4	10.4	12,536	2.8
3-4	636	24.1	21.8	26.4	22,136	19.6	494	20.9	18.6	23.3	12,809	11.3	384	13.5	11.6	15.3	5,166	4.6
5-6	275	40.5	35.0	46.0	4,523	29.6	209	33.0	27.6	38.5	2,418	15.8	124	14.8	11.0	18.7	883	5.8
7-9	62	36.8	26.2	47.4	827	34.3	54	35.3	24.6	45.9	454	18.8	37	23.4	13.9	33.0	182	7.5
10+	83	51.7	40.6	62.8	460	42.4	66	43.0	31.7	54.3	226	20.8	49	28.3	18.3	38.4	120	11.1
** BMI group**																		
Under weight	16	22.8	3.2	42.3	752	13.5	11	9.8	2.7	16.8	539	9.6	8	4.3	0.6	8.0	113	2.0
Normal weight	744	19.3	17.4	21.3	30,049	11.4	702	18.3	16.3	20.3	22,790	8.7	389	8.7	7.4	10.1	6,572	2.5
Over weight	1,151	17.6	16.2	19.0	40,556	10.6	876	15.3	13.9	16.7	25,030	6.5	774	10.6	9.5	11.7	10,702	2.8
Obese	364	18.5	16.1	20.8	14,308	11.4	239	14.6	12.0	17.2	8,195	6.5	254	10.9	9.0	12.7	4,044	3.2
** Education**																		
High school or less	1,521	23.0	21.5	24.5	74,718	14.8	1,407	22.0	20.5	23.6	53,372	10.6	759	11.0	9.8	12.1	15,223	3.0
Some college	377	15.8	13.9	17.7	12,893	10.2	221	9.1	7.6	10.5	5,903	4.7	167	7.2	5.8	8.6	2,724	2.2
Bachelors degree or more	371	6.9	6.0	7.8	6,401	3.1	185	3.8	3.2	4.5	2,947	1.4	478	8.6	7.7	9.6	5,631	2.7
** Race and ethnicity**																		
Non-Hispanic White	1,436	19.8	18.4	21.3	57,752	11.8	1,120	16.9	15.5	18.4	37,279	7.6	891	10.4	9.4	11.5	13,402	2.7
Non-Hispanic Black	236	13.5	11.1	15.8	12,777	9.5	198	13.3	10.7	15.8	8,797	6.5	196	10.2	8.2	12.3	5,510	4.1
Hispanic	343	18.1	15.6	20.6	13,373	10.1	294	17.3	14.6	20.0	9,174	6.9	210	8.9	7.2	10.6	3,175	2.4
Non-Hispanic Other	252	18.2	15.1	21.3	10,829	11.2	209	14.9	12.2	17.6	7,291	7.6	122	8.8	6.7	11.0	1,869	1.9

Abbreviations: HRBS, Health Risk Behavior Survey; PHA, Periodic Health Assessment; CI, confidence interval; No., number; STI, sexually-transmitted infection; BMI, body mass index.

^a^Groups are not mutually exclusive; thus, total of tobacco use types does not equal total survey respondents.

^b^Weighted percent.

^c^PHA MHA question 5b does not allow for assessment of missing data; thus, no response to this question is assumed as 0 drinks on a typical day when drinking.

**Table 3b.** Prevalence of Tobacco Product Use,^a^ HRBS Versus PHA, by Demographic Characteristics, Active Duty U.S. Military, 2018 (cont.)

	**Pipe/Hookah**						**Chewing Tobacco**						**Any Tobacco Use**					
	**HRBS**				**PHA**		**HRBS**				**PHA**		**HRBS**				**PHA**	
			95% CI						95% CI						95% CI			
	No.	%^b^	Lower	Upper	No.	%	No.	%^b^	Lower	Upper	No.	%	No.	%^b^	Lower	Upper	No.	%
**Total**	721	5.2	4.6	5.7	12,515	1.5	1,531	13.4	12.4	14.3	83,044	9.7	5,160	37.8	36.6	39.0	216,265	25.3
** Sex**																		
Male	464	5.1	4.4	5.7	10,041	1.4	1,447	15.7	14.5	16.8	81,887	11.6	4,004	40.4	39.0	41.8	197,361	28.0
Female	257	5.6	4.6	6.6	2,474	1.7	84	2.0	1.5	2.6	1,157	0.8	1,156	24.8	23.0	26.6	18,904	12.7
** Age**																		
17-24	284	7.4	6.1	8.7	6,618	2.2	377	16.3	14.2	18.4	35,366	11.7	1,472	45.7	43.2	48.3	92,109	30.5
25-34	295	4.7	4.0	5.5	4,699	1.3	617	13.0	11.8	14.3	33,549	9.5	1,959	36.1	34.4	37.7	84,634	24.0
35-44	124	2.5	2.0	3.0	1,063	0.6	435	9.6	8.6	10.6	12,263	7.5	1,397	29.4	27.9	30.9	34,616	21.1
45+	18	0.9	0.4	1.4	135	0.4	102	6.1	4.8	7.4	1,866	5.4	332	18.9	16.7	21.1	4,905	14.1
** Service**																		
Army	107	3.6	2.6	4.6	4,912	1.5	353	14.7	12.7	16.8	41,183	12.6	1,014	36.2	33.6	38.7	97,029	29.7
Navy	155	6.3	4.8	7.8	2,001	1.4	277	12.8	10.8	14.8	9,684	6.8	1,102	40.6	38.0	43.3	32,937	23.0
Marine Corps	121	6.7	5.1	8.3	1,322	1.6	393	19.8	17.3	22.3	14,246	17.2	1,030	49.0	46.1	51.9	28,133	34.0
Air Force	277	5.5	4.8	6.2	4,105	1.5	357	8.6	7.7	9.5	16,077	5.8	1,528	31.2	29.7	32.6	53,349	19.2
Coast Guard	61	4.2	2.8	5.6	175	0.8	151	11.8	9.9	13.8	1,854	8.1	486	35.4	32.6	38.2	4,817	20.9
** Rank**																		
Enlisted	594	5.6	5.0	6.3	11,316	1.6	1,220	14.4	13.3	15.5	74,515	10.7	4,276	41.2	39.7	42.6	199,753	28.6
Officer	127	2.9	2.2	3.5	1,199	0.8	311	8.3	7.3	9.4	8,529	5.5	884	20.8	19.3	22.3	16,512	10.6
** STI risk**																		
No	628	5.1	4.5	5.7	10,591	1.3	1,392	13.2	12.2	14.2	78,020	9.8	4,673	37.5	36.3	38.8	199,303	25.0
Yes	48	9.3	5.1	13.4	1,776	4.1	61	16.5	11.0	22.0	4,440	10.2	203	44.9	38.0	51.7	14,555	33.3
** Sleep**																		
5 hours or less	234	6.4	5.2	7.6	2,305	2.4	535	19.1	16.9	21.2	11,615	12.0	1,665	47.7	45.3	50.1	32,761	33.8
5 to less than 7 hours	252	5.4	4.4	6.5	7,083	1.5	547	12.9	11.3	14.5	47,563	10.2	1,796	36.5	34.5	38.6	123,215	26.5
7-9 hours	222	3.7	2.9	4.5	3,003	1.1	426	9.1	7.9	10.3	23,486	8.3	1,584	30.3	28.4	32.3	58,935	20.8
9 hours or more	13	6.7	2.0	11.3	124	1.8	23	8.9	3.9	14.0	369	5.3	115	35.0	27.0	43.1	1,336	19.1
** Alcohol use^c^**																		
No response / none	87	2.5	1.7	3.3	2,618	1.0	242	8.6	7.0	10.2	19,815	7.4	821	25.0	22.6	27.3	50,791	19.0
1-2	291	4.4	3.6	5.2	6,079	1.3	619	11.1	9.8	12.4	40,881	9.0	2,194	32.7	31.0	34.3	108,266	23.8
3-4	217	7.3	5.9	8.8	3,115	2.8	415	17.2	15.1	19.3	17,833	15.8	1,415	52.1	49.5	54.8	46,895	41.5
5-6	70	8.2	5.7	10.8	520	3.4	170	28.4	22.9	33.8	3,518	23.0	487	63.5	58.5	68.6	8,216	53.8
7-9	16	11.8	3.6	20.0	112	4.6	36	21.6	11.9	31.3	660	27.4	117	67.2	56.8	77.6	1,421	58.9
10+	40	21.2	12.2	30.1	71	6.5	49	31.4	20.7	42.1	337	31.1	126	72.2	63.1	81.3	676	62.3
** BMI group**																		
Under weight	5	3.3	0.0	6.8	96	1.7	10	27.6	4.5	50.7	419	7.5	33	44.3	23.7	64.8	1,441	25.8
Normal weight	267	5.8	4.7	6.8	4,129	1.6	400	12.2	10.4	13.9	21,539	8.2	1,583	36.7	34.4	38.9	63,848	24.3
Over weight	352	4.8	4.0	5.5	5,239	1.4	864	13.7	12.5	15.0	39,046	10.2	2,732	38.0	36.4	39.7	96,196	25.1
Obese	97	5.2	3.7	6.7	1,859	1.5	257	14.5	11.9	17.1	12,711	10.1	812	39.5	36.4	42.5	33,339	26.6
** Education**																		
High school or less	445	6.3	5.4	7.1	9,189	1.8	935	15.9	14.5	17.3	60,910	12.1	3,136	44.4	42.7	46.2	160,107	31.7
Some college	90	3.3	2.5	4.2	1,452	1.1	220	11.1	9.3	12.9	9,985	7.9	799	32.7	30.4	35.1	28,613	22.6
Bachelors degree or more	176	3.1	2.6	3.7	1,738	0.8	366	7.6	6.7	8.6	11,331	5.4	1,182	22.2	20.8	23.6	25,453	12.2
** Race and ethnicity**																		
Non-Hispanic White	355	4.3	3.6	5.0	5,177	1.1	1,195	17.7	16.3	19.1	65,104	13.3	3,347	41.2	39.6	42.8	139,480	28.4
Non-Hispanic Black	151	8.0	6.1	9.9	4,032	3.0	67	4.3	2.9	5.6	3,856	2.9	557	29.4	26.3	32.5	27,546	20.4
Hispanic	132	5.9	4.3	7.4	2,032	1.5	134	8.6	6.6	10.6	7,139	5.4	708	34.8	31.7	37.9	26,711	20.1
Non-Hispanic Other	78	4.6	3.3	5.8	1,274	1.3	133	11.5	8.7	14.4	6,945	7.2	531	36.8	33.2	40.4	22,528	23.4

Abbreviations: HRBS, Health Risk Behavior Survey; PHA, Periodic Health Assessment; CI, confidence interval; No., number; STI, sexually-transmitted infection; BMI, body mass index.

^a^Groups are not mutually exclusive; thus, total of tobacco use types does not equal total survey respondents.

^b^Weighted percent.

^c^PHA MHA question 5b does not allow for assessment of missing data; thus, no response to this question is assumed as 0 drinks on a typical day when drinking.

**Table 3c.** Prevalence of Tobacco Product Use,^a^ HRBS Versus PHA, by Demographic Characteristics, Active Duty U.S. Military, 2018 (cont.)

	**Two or More Tobacco Products**					
	**HRBS**				**PHA**	
			95% CI			
	No.	%^b^	Lower	Upper	No.	%
**Total**	1,886	17.4	16.3	18.5	49,768	5.8
** Sex**						
Male	1,511	19.1	17.8	20.3	46,610	6.6
Female	375	9.0	7.7	10.2	3,158	2.1
** Age**						
17-24	731	25.7	23.3	28.0	29,548	9.8
25-34	716	15.1	13.7	16.5	15,563	4.4
35-44	380	8.3	7.3	9.2	4,293	2.6
45+	59	3.6	2.5	4.6	364	1.0
** Service**						
Army	329	16.1	13.9	18.2	24,625	7.5
Navy	417	20.2	17.8	22.6	6,205	4.3
Marine Corps	426	26.0	23.2	28.8	8,376	10.1
Air Force	535	11.9	10.8	12.9	9,878	3.5
Coast Guard	179	14.1	11.8	16.3	684	3.0
** Rank**						
Enlisted	1,660	19.7	18.4	20.9	47,952	6.9
Officer	226	5.7	4.8	6.6	1,816	1.2
** STI risk**						
No	1,660	16.9	15.7	18.0	44,444	5.6
Yes	108	26.2	19.8	32.7	4,801	11.0
** Sleep**						
5 hours or less	698	24.9	22.6	27.3	9,409	9.7
5 to less than 7 hours	644	15.8	14.1	17.4	28,731	6.2
7-9 hours	507	12.3	10.7	13.8	11,267	4.0
9 hours or more	37	16.1	9.6	22.6	359	5.1
** Alcohol use^c^**						
No response / none	297	11.0	9.1	12.8	14,158	5.3
1-2	663	12.6	11.3	13.9	20,145	4.4
3-4	544	23.0	20.6	25.4	11,775	10.4
5-6	241	38.1	32.6	43.6	2,782	18.2
7-9	57	41.1	30.0	52.3	573	23.8
10+	84	58.0	47.5	68.5	335	30.9
** BMI group**						
Under weight	15	21.0	1.5	40.5	363	6.5
Normal weight	636	18.4	16.4	20.3	16,663	6.3
Over weight	947	16.9	15.5	18.4	20,587	5.4
Obese	288	16.4	13.9	18.9	6,770	5.4
** Education**						
High school or less	1,337	22.6	21.0	24.1	42,163	8.4
Some college	223	11.4	9.5	13.3	4,298	3.4
Bachelors degree or more	311	6.2	5.3	7.0	3,009	1.4
** Race and ethnicity**						
Non-Hispanic White	1,190	18.8	17.3	20.2	32,382	6.6
Non-Hispanic Black	210	13.6	11.1	16.1	6,057	4.5
Hispanic	285	17.2	14.5	19.8	6,495	4.9
Non-Hispanic Other	195	16.0	12.8	19.1	4,834	5.0

Abbreviations: HRBS, Health Risk Behavior Survey; PHA, Periodic Health Assessment; CI, confidence interval; No., number; STI, sexually-transmitted infection; BMI, body mass index.

^a^Groups are not mutually exclusive; thus, total of tobacco use types does not equal total survey respondents.

^b^Weighted percent.

^c^PHA MHA question 5b does not allow for assessment of missing data; thus, no response to this question is assumed as 0 drinks on a typical day when drinking.

**Table 4a.** Adjusted Odds Ratios (95% CI) for Tobacco Use by Product in the Active Duty U.S. Military, HRBS Versus PHA, 2018

	**Cigarettes**		**E-Cigarettes**		**Cigars**	
	**HRBS**	**PHA**	**HRBS**	**PHA**	**HRBS**	**PHA**
**Sex**						
Male	Ref	Ref	Ref	Ref	Ref	Ref
Female	0.77 (0.65, 0.90)	0.74 (0.72, 0.75)	0.72 (0.60, 0.87)	0.58 (0.56, 0.59)	0.44 (0.35, 0.55)	0.30 (0.28, 0.31)
**Age**						
17-24	Ref	Ref	Ref	Ref	Ref	Ref
25-34	0.94 (0.78, 1.14)	0.96 (0.94, 0.97)	0.40 (0.33, 0.49)	0.50 (0.49, 0.51)	0.74 (0.58, 0.93)	0.58 (0.56,0.60)
35-44	0.85 (0.69, 1.04)	1.14 (1.12, 1.17)	0.23 (0.18, 0.30)	0.37 (0.36, 0.38)	0.63 (0.49, 0.81)	0.59 (0.56, 0.62)
45+	0.63 (0.46, 0.85)	0.88 (0.83, 0.93)	0.11 (0.07, 0.18)	0.20 (0.18, 0.23)	0.55 (0.39, 0.76)	0.70 (0.65, 0.75)
**Service**						
Army	Ref	Ref	Ref	Ref	Ref	Ref
Navy	0.99 (0.79, 1.24)	0.68 (0.66, 0.69)	1.05 (0.80, 1.38)	1.16 (1.12, 1.19)	1.34 (1.01, 1.78)	0.85 (0.81, 0.89)
Marine Corps	1.19 (0.94, 1.50)	0.88 (0.86, 0.90)	0.96 (0.73, 1.26)	0.96 (0.93, 0.99)	1.32 (0.99, 1.77)	1.09 (1.04, 1.14)
Air Force	0.69 (0.57, 0.83)	0.48 (0.47, 0.49)	1.21 (0.98, 1.51)	1.15 (1.13, 1.18)	0.88 (0.69, 1.12)	0.92 (0.89, 0.95)
Coast Guard	0.69 (0.54, 0.88)	0.38 (0.36, 0.40)	1.23 (0.93, 1.63)	1.00 (0.94, 1.07)	1.31 (0.98, 1.77)	1.11 (1.01, 1.21)
**Rank**						
Enlisted	Ref	Ref	Ref	Ref	Ref	Ref
Officer	0.44 (0.34, 0.56)	0.21 (0.20, 0.23)	0.28 (0.20, 0.40)	0.22 (0.20, 0.24)	1.15 (0.90, 1.46)	1.33 (1.25, 1.41)
**STI risk**						
No	Ref	Ref	Ref	Ref	Ref	Ref
Yes	1.22 (0.85, 1.75)	1.46 (1.41, 1.50)	1.47 (1.01, 2.14)	1.50 (1.45, 1.55)	1.15 (0.68, 1.94)	1.64 (1.57, 1.73)
**Sleep**						
5 hours or less	1.56 (1.30, 1.86)	1.44 (1.41, 1.47)	1.31 (1.07, 1.62)	1.32 (1.28, 1.35)	1.28 (1.02, 1.60)	1.30 (1.25, 1.35)
5 to less than 7 hours	Ref	Ref	Ref	Ref	Ref	Ref
7-9 hours	0.88 (0.73, 1.07)	0.79 (0.77, 0.80)	0.79 (0.63, 0.97)	0.73 (0.71, 0.74)	0.89 (0.72, 1.11)	0.76 (0.74, 0.79)
9 hours or more	0.96 (0.59, 1.57)	0.85 (0.78, 0.92)	0.89 (0.55, 1.45)	0.68 (0.62, 0.76)	0.57 (0.29, 1.12)	0.79 (0.67, 0.94)
**Alcohol use^a^**						
No response / none	Ref	Ref	Ref	Ref	Ref	Ref
1-2	1.36 (1.10, 1.68)	1.64 (1.61, 1.67)	1.23 (0.97, 1.56)	1.26 (1.23, 1.28)	2.10 (1.54, 2.86)	1.70 (1.64, 1.76)
3-4	2.39 (1.90, 3.00)	2.68 (2.62, 2.74)	2.16 (1.68, 2.79)	1.83 (1.78, 1.87)	2.74 (1.96, 3.82)	2.40 (2.30, 2.50)
5-6	3.91 (2.88, 5.30)	3.67 (3.52, 3.83)	3.09 (2.22, 4.29)	2.11 (2.00, 2.22)	2.76 (1.79, 4.24)	2.61 (2.41, 2.83)
7-9	3.30 (1.95, 5.60)	3.93 (3.57, 4.33)	3.27 (1.76, 6.08)	2.24 (2.00, 2.52)	3.78 (1.98, 7.23)	3.01 (2.54, 3.58)
10+	5.77 (3.46, 9.61)	5.09 (4.43, 5.84)	4.19 (2.44, 7.20)	2.08 (1.75, 2.47)	5.43 (3.00, 9.84)	4.22 (3.38, 5.26)
**BMI group**						
Under weight	1.14 (0.48, 2.67)	1.17 (1.08, 1.27)	0.38 (0.12, 1.22)	1.08 (0.99, 1.19)	0.42 (0.13, 1.32)	0.85 (0.70, 1.03)
Normal weight	Ref	Ref	Ref	Ref	Ref	Ref
Over weight	0.86 (0.72, 1.03)	0.85 (0.84, 0.87)	1.00 (0.82, 1.21)	0.84 (0.82, 0.86)	1.22 (0.97, 1.52)	1.07 (1.04, 1.11)
Obese	0.86 (0.69, 1.09)	0.87 (0.85, 0.89)	0.98 (0.73, 1.31)	0.89 (0.86, 0.91)	1.21 (0.91, 1.62)	1.25 (1.20, 1.30)
**Education**						
High school or less	Ref	Ref	Ref	Ref	Ref	Ref
Some college	0.82 (0.68, 0.99)	0.68 (0.66, 0.69)	0.74 (0.58, 0.93)	0.64 (0.62, 0.66)	0.82 (0.64, 1.05)	0.89 (0.85, 0.93)
Bachelors degree or more	0.51 (0.41, 0.64)	0.38 (0.37, 0.40)	0.65 (0.49, 0.85)	0.43 (0.41, 0.45)	0.98 (0.76, 1.25)	0.99 (0.93, 1.05)
**Race and ethnicity**						
Non-Hispanic White	Ref	Ref	Ref	Ref	Ref	Ref
Non-Hispanic Black	0.58 (0.45, 0.74)	0.61 (0.60, 0.63)	0.71 (0.54, 0.94)	0.75 (0.73, 0.77)	1.17 (0.89, 1.56)	1.69 (1.63, 1.75)
Hispanic	0.73 (0.59, 0.90)	0.68 (0.66, 0.69)	0.81 (0.64, 1.03)	0.74 (0.72, 0.76)	0.80 (0.63, 1.03)	0.90 (0.86, 0.94)
Non-Hispanic Other	0.88 (0.69, 1.14)	0.97 (0.95, 1.00)	0.89 (0.68, 1.17)	1.02 (0.99, 1.05)	0.89 (0.65, 1.22)	0.77 (0.73, 0.82)

Abbreviations: CI, confidence interval; HRBS, Health Risk Behavior Survey; PHA, Periodic Health Assessment; e-cigarette, electronic cigarette; STI, sexually-transmitted infection; BMI, body mass index.

^a^PHA MHA question 5b does not allow for assessment of missing data; thus, no response to this question is assumed as 0 drinks on a typical day when drinking.

Note: These analyses used weighted (HRBS) and unweighted (PHA) logistic regression.

**Table 4b.** Adjusted Odds Ratios (95% CI) for Tobacco Use by Product in the Active Duty U.S. Military, HRBS Versus PHA, 2018 (cont.)

	**Pipe/Hookah**		**Chewing Tobacco**		**Any Tobacco Use**	
	**HRBS**	**PHA**	**HRBS**	**PHA**	**HRBS**	**PHA**
**Sex**						
Male	Ref	Ref	Ref	Ref	Ref	Ref
Female	1.14 (0.88, 1.47)	1.09 (1.04, 1.14)	0.14 (0.10, 0.19)	0.09 (0.08, 0.09)	0.58 (0.51, 0.66)	0.47 (0.46, 0.48)
**Age**						
17-24	Ref	Ref	Ref	Ref	Ref	Ref
25-34	0.62 (0.47, 0.83)	0.59 (0.57, 0.62)	0.87 (0.70, 1.09)	0.92 (0.90, 0.94)	0.75 (0.64, 0.87)	0.85 (0.84, 0.86)
35-44	0.33 (0.23, 0.46)	0.30 (0.27, 0.32)	0.65 (0.51, 0.83)	0.75 (0.72, 0.77)	0.62 (0.53, 0.74)	0.84 (0.83, 0.86)
45+	0.15 (0.08, 0.28)	0.21 (0.18, 0.26)	0.45 (0.32, 0.64)	0.59 (0.55, 0.62)	0.46 (0.37, 0.57)	0.69 (0.67, 0.72)
**Service**						
Army	Ref	Ref	Ref	Ref	Ref	Ref
Navy	1.60 (1.07, 2.40)	1.02 (0.96, 1.08)	0.72 (0.55, 0.94)	0.54 (0.52, 0.55)	0.98 (0.82, 1.17)	0.73 (0.72, 0.74)
Marine Corps	1.48 (0.96, 2.27)	0.94 (0.88, 1.01)	0.93 (0.72, 1.21)	1.23 (1.20, 1.26)	1.13 (0.93, 1.36)	1.01 (0.99, 1.02)
Air Force	1.79 (1.27, 2.54)	1.25 (1.20, 1.31)	0.56 (0.45, 0.69)	0.46 (0.45, 0.47)	0.83 (0.72, 0.96)	0.63 (0.62, 0.64)
Coast Guard	1.53 (0.91, 2.57)	0.69 (0.58, 0.82)	0.69 (0.52, 0.90)	0.51 (0.48, 0.54)	0.85 (0.71, 1.03)	0.55 (0.53, 0.57)
**Rank**						
Enlisted	Ref	Ref	Ref	Ref	Ref	Ref
Officer	0.88 (0.63, 1.22)	0.85 (0.77, 0.94)	1.00 (0.77, 1.30)	0.81 (0.78, 0.84)	0.64 (0.54, 0.75)	0.54 (0.52, 0.55)
**STI risk**						
No	Ref	Ref	Ref	Ref	Ref	Ref
Yes	1.37 (0.75, 2.47)	2.22 (2.10, 2.35)	1.68 (1.05, 2.67)	1.05 (1.01, 1.09)	1.31 (0.95, 1.80)	1.38 (1.35, 1.42)
**Sleep**						
5 hours or less	1.03 (0.76, 1.40)	1.41 (1.34, 1.49)	1.49 (1.21, 1.85)	1.17 (1.14, 1.20)	1.44 (1.25, 1.67)	1.32 (1.30, 1.34)
5 to less than 7 hours	Ref	Ref	Ref	Ref	Ref	Ref
7-9 hours	0.67 (0.49, 0.91)	0.75 (0.72, 0.79)	0.74 (0.59, 0.91)	0.87 (0.85, 0.89)	0.81 (0.71, 0.94)	0.81 (0.80, 0.82)
9 hours or more	1.07 (0.48, 2.39)	1.00 (0.83, 1.20)	0.61 (0.31, 1.22)	0.59 (0.52, 0.66)	0.95 (0.64, 1.39)	0.70 (0.65, 0.75)
**Alcohol use^a^**						
No response / none	Ref	Ref	Ref	Ref	Ref	Ref
1-2	2.31 (1.55, 3.44)	1.90 (1.81, 2.00)	1.44 (1.11, 1.87)	1.55 (1.52, 1.58)	1.76 (1.51, 2.06)	1.83 (1.81, 1.86)
3-4	3.58 (2.36, 5.42)	3.25 (3.07, 3.44)	2.08 (1.59, 2.73)	2.16 (2.11, 2.22)	3.48 (2.92, 4.15)	3.13 (3.08, 3.19)
5-6	4.08 (2.48, 6.70)	3.49 (3.14, 3.88)	2.89 (2.01, 4.16)	2.71 (2.59, 2.84)	4.32 (3.30, 5.65)	4.08 (3.93, 4.24)
7-9	4.89 (1.87, 12.79)	4.27 (3.44, 5.31)	1.79 (0.92, 3.50)	3.05 (2.75, 3.39)	4.34 (2.55, 7.38)	4.28 (3.90, 4.70)
10+	12.24 (6.31, 23.75)	6.01 (4.57, 7.91)	2.97 (1.73, 5.11)	3.24 (2.79, 3.78)	5.74 (3.47, 9.48)	4.57 (3.97, 5.27)
**BMI group**						
Under weight	0.44 (0.11, 1.82)	1.06 (0.86, 1.31)	3.29 (1.19, 9.05)	0.96 (0.87, 1.07)	1.33 (0.62, 2.85)	1.09 (1.02, 1.16)
Normal weight	Ref	Ref	Ref	Ref	Ref	Ref
Over weight	0.92 (0.69, 1.22)	0.97 (0.93, 1.02)	1.02 (0.83, 1.26)	1.19 (1.16, 1.21)	1.04 (0.91, 1.19)	0.99 (0.97, 1.00)
Obese	0.97 (0.66, 1.42)	1.04 (0.98, 1.10)	1.22 (0.90, 1.66)	1.36 (1.33, 1.39)	1.08 (0.90, 1.30)	1.07 (1.05, 1.09)
**Education**						
High school or less	Ref	Ref	Ref	Ref	Ref	Ref
Some college	0.83 (0.59, 1.17)	0.88 (0.82, 0.93)	0.79 (0.62, 1.00)	0.74 (0.72, 0.76)	0.77 (0.66, 0.90)	0.67 (0.66, 0.69)
Bachelors degree or more	1.01 (0.74, 1.38)	0.86 (0.78, 0.93)	0.56 (0.43, 0.73)	0.51 (0.49, 0.53)	0.62 (0.53, 0.73)	0.44 (0.43, 0.45)
**Race and ethnicity**						
Non-Hispanic White	Ref	Ref	Ref	Ref	Ref	Ref
Non-Hispanic Black	2.34 (1.68, 3.25)	2.69 (2.57, 2.82)	0.19 (0.13, 0.28)	0.18 (0.17, 0.18)	0.56 (0.46, 0.67)	0.55 (0.54, 0.56)
Hispanic	1.25 (0.88, 1.78)	1.32 (1.25, 1.39)	0.37 (0.27, 0.49)	0.32 (0.31, 0.33)	0.60 (0.51, 0.71)	0.52 (0.51, 0.52)
Non-Hispanic Other	1.12 (0.78, 1.61)	1.26 (1.17, 1.34)	0.63 (0.47, 0.86)	0.57 (0.55, 0.58)	0.83 (0.69, 1.00)	0.79 (0.78, 0.80)

Abbreviations: CI, confidence interval; HRBS, Health Risk Behavior Survey; PHA, Periodic Health Assessment; e-cigarette, electronic cigarette; STI, sexually-transmitted infection; BMI, body mass index.

^a^PHA MHA question 5b does not allow for assessment of missing data; thus, no response to this question is assumed as 0 drinks on a typical day when drinking.

Note: These analyses used weighted (HRBS) and unweighted (PHA) logistic regression.

**Table 4c.** Adjusted Odds Ratios (95% CI) for Tobacco Use by Product in the Active Duty U.S. Military, HRBS Versus PHA, 2018 (cont.)

	**Two or More Tobacco Products**	
	**HRBS**	**PHA**
**Sex**						
Male	Ref	Ref
Female	0.48 (0.39, 0.58)	0.38 (0.36, 0.39)
**Age**						
17-24	Ref	Ref
25-34	0.62 (0.51, 0.76)	0.56 (0.54, 0.57)
35-44	0.36 (0.29, 0.45)	0.39 (0.37, 0.40)
45+	0.20 (0.14, 0.30)	0.21 (0.19, 0.24)
**Service**						
Army	Ref	Ref
Navy	1.13 (0.89, 1.45)	0.66 (0.64, 0.68)
Marine Corps	1.07 (0.83, 1.38)	1.02 (0.99, 1.05)
Air Force	0.80 (0.65, 0.99)	0.57 (0.55, 0.58)
Coast Guard	0.93 (0.71, 1.22)	0.45 (0.42, 0.49)
**Rank**						
Enlisted	Ref	Ref
Officer	0.64 (0.50, 0.83)	0.47 (0.44, 0.51)
**STI risk**						
No	Ref	Ref
Yes	1.65 (1.13, 2.42)	1.76 (1.70, 1.83)
**Sleep**						
5 hours or less	1.50 (1.24, 1.83)	1.50 (1.46, 1.54)
5 to less than 7 hours	Ref	Ref
7-9 hours	0.80 (0.65, 0.99)	0.68 (0.66, 0.69)
9 hours or more	0.88 (0.51, 1.52)	0.73 (0.65, 0.82)
**Alcohol use^a^**						
No response / none	Ref	Ref
1-2	1.53 (1.20, 1.94)	1.36 (1.33, 1.40)
3-4	2.67 (2.08, 3.44)	2.23 (2.16, 2.29)
5-6	4.16 (3.02, 5.74)	3.05 (2.90, 3.21)
7-9	4.09 (2.31, 7.26)	3.54 (3.17, 3.94)
10+	8.51 (5.17, 14.02)	4.14 (3.56, 4.82)
**BMI group**						
Under weight	1.01 (0.41, 2.49)	1.02 (0.91, 1.14)
Normal weight	Ref	Ref
Over weight	0.98 (0.81, 1.18)	0.91 (0.89, 0.93)
Obese	0.95 (0.72, 1.24)	1.02 (0.98, 1.05)
**Education**						
High school or less	Ref	Ref
Some college	0.75 (0.59, 0.95)	0.62 (0.60, 0.64)
Bachelors degree or more	0.55 (0.44, 0.70)	0.41 (0.39, 0.44)
**Race and ethnicity**						
Non-Hispanic White	Ref	Ref
Non-Hispanic Black	0.68 (0.52, 0.89)	0.57 (0.56, 0.59)
Hispanic	0.73 (0.58, 0.92)	0.59 (0.57, 0.61)
Non-Hispanic Other	0.84 (0.63, 1.11)	0.83 (0.80, 0.86)

Abbreviations: CI, confidence interval; HRBS, Health Risk Behavior Survey; PHA, Periodic Health Assessment; e-cigarette, electronic cigarette; STI, sexually-transmitted infection; BMI, body mass index.

^a^PHA MHA question 5b does not allow for assessment of missing data; thus, no response to this question is assumed as 0 drinks on a typical day when drinking.

Note: These analyses used weighted (HRBS) and unweighted (PHA) logistic regression.

## AUTHOR AFFILIATIONS

Uniformed Services University Department of Preventive Medicine and Biostatistics: Dr. Mancuso and Dr. Ahmed; Armed Forces Health Surveillance Division: Ms. Rossi
